# Case‐control study of heart rate abnormalities across the breast cancer survivorship continuum

**DOI:** 10.1002/cam4.1916

**Published:** 2018-12-21

**Authors:** John D. Groarke, Syed S. Mahmood, David Payne, Sarju Ganatra, Jon Hainer, Tomas G. Neilan, Ann H. Partridge, Marcelo F. Di Carli, Lee W. Jones, Mandeep R. Mehra, Anju Nohria

**Affiliations:** ^1^ Heart and Vascular Center Brigham and Women’s Hospital Boston Massachusetts; ^2^ Department of Radiology Brigham and Women's Hospital Boston Massachusetts; ^3^ Adult Survivorship Program Dana‐Farber Cancer Institute and Brigham and Women’s Hospital Boston Massachusetts; ^4^ Cardio‐Oncology Program Massachusetts General Hospital Boston Massachusetts; ^5^ Department of Medicine Memorial Sloan Kettering Cancer Center New York City New York

**Keywords:** abnormal heart rate recovery, breast cancer, cancer survivorship, elevated resting heart rate, exercise capacity

## Abstract

**Background:**

Mechanisms underlying impaired exercise capacity and increased cardiovascular mortality observed in breast cancer (BC) patients remain unclear. The prevalence, functional, and prognostic significance of elevated resting heart rate (HR) and abnormal heart rate recovery (HRR) in breast cancer (BC) requires evaluation.

**Methods:**

In a single‐center, retrospective, case‐control study of women referred for exercise treadmill testing (ETT), 448 BC patients (62.6 ± 10.0 years) were compared to 448 cancer‐free, age‐matched controls. Elevated resting HR was defined as HR ≥80 bpm at rest. Abnormal HRR at 1‐minute following exercise was defined as ≤12 bpm if active recovery or ≤18 bpm if passive recovery. Association of these parameters with exercise capacity and all‐cause mortality was evaluated.

**Results:**

Elevated resting HR (23.7% vs 17.0%, *P* = 0.013) and abnormal HRR (25.9% vs 20.3%, *P* = 0.048) were more prevalent in BC cohort than controls. In adjusted analyses, BC patients with elevated resting HR (−0.9 METs (SE 0.3); *P* = 0.0003) or abnormal HRR (−1.3 METs (SE 0.3); *P* < 0.0001) had significant reductions in metabolic equivalents (METs) achieved during exercise. Elevated resting HR was not associated with mortality. There was a trend toward increased mortality in BC cohort with abnormal HRR (adjusted hazard ratio 2.06 (95% CI 0.95‐4.44, *P* = 0.07)).

**Conclusions:**

Women across the BC survivorship continuum, referred for ETT, have an increased prevalence of elevated resting HR and abnormal HRR relative to cancer‐free, age‐matched female controls. These parameters were associated with decreased exercise capacity. Strategies to modulate these abnormalities may help improve functional capacity in this cohort.

## 1 INTRODUCTION

Medical advances have significantly decreased breast cancer (BC) mortality.^1^, ^2^ Consequently, the population of BC survivors in the United States is projected to increase to 3.9 million by 2024.^2^ BC survivors are at increased risk of death from cardiovascular disease, and cardiovascular death is the leading cause of mortality among older BC survivors.^3^, ^4^, ^5^ Exercise capacity is an independent predictor of cardiovascular and all‐cause mortality among asymptomatic women in the general population,^6^ and exercise capacity is impaired in women across the BC survivorship continuum.^7^ Mechanisms leading to decreased exercise capacity and increased mortality in BC survivors need to be explored.

Elevated resting heart rate (HR) and abnormal heart rate recovery (HRR) after exercise are established markers of autonomic dysfunction^8^ that are associated with increased cardiovascular and all‐cause mortality in the general population.^9^, ^10^, ^11^, ^12^, ^13^, ^14^ Patients with early‐stage BC manifest higher resting HRs than age‐matched controls.^7^ Elevated resting HR is associated with increased all‐cause mortality in patients with active BC,^15^ while abnormal HRR is associated with worse survival in other cancers.^16^, ^17^, ^18^ We have previously shown that elevated resting HR and abnormal HRR are associated with reduced exercise capacity in survivors of Hodgkin lymphoma, treated with mediastinal radiation therapy.^18^ Therefore, we hypothesized that elevated resting HR and abnormal HRR are prevalent and contribute to exercise limitation and mortality across the BC survivorship continuum.

We evaluated the prevalence, and functional and prognostic significance of elevated resting HR and abnormal HRR among women clinically referred for exercise treadmill tests (ETT) at our institution, following a diagnosis of BC.

## 2 METHODS

### 2.1 Study design

In this retrospective, case‐control study, we identified 520 consecutive women with a history of BC who were clinically referred to Brigham and Women's Hospital for ETT between 05/03/2002 and 08/13/2015. Oncology records were unavailable for 72 women, resulting in a final cohort of 448 BC patients. Cancer‐free, age‐ and sex‐matched controls were identified for each case. This study complies with the declaration of Helsinki and was approved by the Partners Healthcare Institutional Review Board.

### 2.2 Demographics, cardiovascular, and cancer treatment history

Demographics, cardiovascular risk factors, medications, and symptoms were collected at the time of ETT by a structured patient interview and medical chart review. Ischemic heart disease was defined as prior myocardial infarction, coronary revascularization, or documented angiographic coronary artery disease. Congestive heart failure included history of heart failure, cardiomyopathy, or loop diuretic use. Hyperlipidemia was defined as history of hyperlipidemia or statin use. Diabetes was defined as history of diabetes or use of insulin or oral hypoglycemic agents. A positive smoking history included ongoing or prior tobacco use. The Morise score was calculated for each patient to assess the pre‐test probability of a positive stress test. This score considers age, sex, smoking, hyperlipidemia, diabetes, hypertension, estrogen status, body mass index, family history of coronary artery disease, and symptoms.^19^

Oncologic history including year of BC diagnosis, laterality, stage at diagnosis, receptor status, specific cancer treatment, interval from diagnosis to ETT, and status of BC at the time of ETT were collected from medical records.

### 2.3 Exercise protocol

All patients exercised according to the standard Bruce Protocol.^20^ HR and blood pressure (BP) were recorded at rest, after each 3‐minute stage of exercise, at maximum exercise, and at 1, 3, and 5 minutes in recovery. Exercise was continued until ≥1 of the following endpoints: exhaustion, symptom limitation, ≥85% of age‐predicted maximal HR (APMHR = 220‐age), ≥10 mm Hg drop in systolic BP from baseline, sustained ventricular tachycardia, ST‐segment depression ≥3 mm measured 80 ms after the J junction, or ST‐segment elevation ≥1 mm. ETTs performed as part of stress myocardial perfusion imaging (n = 418) involved a 1 minute active cooldown at 1.5 miles/hour recovery protocol, whereas ETTs performed in isolation (n = 322) or during stress echocardiography (n = 156) involved passive recovery in the supine position. ETTs were performed, analyzed and reported per international standards^21^ using a computerized database. Resting left ventricular ejection fraction (LVEF) was recorded for ETTs performed with echocardiography or nuclear myocardial perfusion imaging.

### 2.4 Study end‐points

Primary study end‐points included (a) elevated resting HR and (b) abnormal HRR. Elevated resting HR was defined as HR ≥80 beats/min (bpm) at rest.^10^, ^13^, ^14^ Abnormal HRR was defined as the difference between HR at peak exercise and HR at 1 minute into recovery following cessation of exercise: ≤12 bpm for ETTs involving active cooldown or ≤18 bpm for ETTs involving passive recovery.^11^, ^22^, ^23^

The secondary end‐point was workload achieved during exercise in metabolic equivalents (METs).^24^

### 2.5 Follow‐up and all‐cause mortality

All‐cause and cause‐specific mortality through December 2015 were determined using the National Death Index. Median follow‐up was 3.5 [1.9, 5.4] years after the index ETT.

### 2.6 Statistical analyses

Categorical variables are presented as percentages and compared using Fisher's exact test. Continuous, normally distributed variables are presented as mean ± standard deviation (SD), and compared using Student's *t*‐test. Continuous, non‐normal data are presented as median with interquartile range (IQR) and compared using Wilcoxon rank sum test. Data were complete other than for LVEF which was available for 278 women in breast cancer cohort and 208 women in control cohort; LVEF was not included in multivariable models. Multivariable linear regression was used to examine the relationship between study end‐points and METs achieved during ETT after controlling for age, BMI, cardiovascular risk factors, statins, atrioventricular (AV) nodal blocking agents, and result of ETT. Logistic regression was used to examine the relationship between the study end‐points and BC diagnosis, adjuvant chemotherapy, and exposure to anthracyclines, radiation, and hormone therapy. Multivariable logistic regression was used to control for aggregate confounding by Morise score, AV nodal blocking agents, and result of ETT. Unadjusted and adjusted odds ratios (OR) with 95% confidence intervals (CI) are presented. Cox proportional hazards regression models were used to estimate risk of all‐cause mortality with study end‐points; models were adjusted for age, AV nodal blocking agents, Morise risk score, and result of ETT. A linear interaction term for breast cancer diagnosis and each end‐point was included in regression analyses. Unadjusted and adjusted hazard ratios with 95% CI are presented. To determine whether alternative definitions of elevated resting HR would yield different results, a sensitivity analysis using (a) an arbitrary 3‐level categorization of resting HR <80 bpm, 80‐89 bpm, and ≥90 bpm, and (b) a tertile‐based 3‐level categorization of resting HR <65 bpm, 65‐74 bpm, and ≥75 bpm was performed for regression analyses exploring associations with exercise capacity and all‐cause mortality. A two‐sided *P* value of 0.05 was considered significant. Statistical analyses were performed using SAS version 9.4 (SAS Institute, Cary, NC, USA).

## 3 RESULTS

### 3.1 Baseline characteristics

Baseline characteristics are shown in Table 1. BC patients were leaner with a lower prevalence of hyperlipidemia, diabetes, smoking, and known ischemic heart disease relative to controls. Among those with LVEF assessment at the time of ETT (n = 486), mean LVEF was normal, but lower, in the BC cohort. BC patients had a lower frequency of aspirin and statin use. Importantly, use of AV nodal blocking agents was similar in both groups.

**Table 1:** Baseline characteristics of the study population

Characteristic	Breast cancer (n = 448)	Controls (n = 448)	*P* value
Age, mean (SD), y	62.6 ± 10.0	62.5 ± 10.0	0.92
BMI, mean (SD), kg/m^2^	27.0 ± 5.2	28.8 ± 6.3	0.0001
Cardiovascular history			
Hypertension, n (%)	229 (51.1%)	254 (56.7%)	0.11
Hyperlipidemia, n (%)	221 (49.3%)	265 (59.2%)	0.004
Diabetes mellitus, n (%)	49 (10.9%)	86 (19.2%)	0.0007
Ischemic heart disease, n (%)	39 (8.7%)	61 (13.6%)	0.03
Smoking history, n (%)	23 (5.1%)	39 (8.7%)	0.05
Congestive heart failure, n (%)	28 (6.3%)	21 (4.7%)	0.38
LVEF^a^, mean (SD), %	64.4 ± 9.8	66.9 ± 9.3	0.004
Morise risk score, median (IQR)	13.5 (11.0, 16.0)	13.5 (11.0, 16.0)	0.66
Cardiovascular medications			
Beta‐blocker, n (%)	143 (31.9%)	161 (35.9%)	0.23
Calcium channel blocker, n (%)	60 (13.4%)	78 (17.4%)	0.12
ACE/ARB, n (%)	88 (19.6%)	99 (22.1%)	0.41
Aspirin, n (%)	148 (33.0%)	178 (39.7%)	0.04
Statin, n (%)	167 (37.3%)	202 (45.1%)	0.02

ACE/ARB, angiotensin converting enzyme inhibitor/angiotensin receptor blocker; BMI, body mass index; LVEF, left ventricular ejection fraction.

a Available for 278 women in breast cancer cohort and 208 women in control cohort.

### 3.2 Oncology history

Oncologic details are provided in Table S1. Staging data were available for 348 (77.7%) BC patients, with 93.1% having ≤stage II disease at the time of diagnosis. Active malignancy was present in 7.8% at the time of ETT. Cancer treatment included surgery, adjuvant radiation therapy, chemotherapy, and hormone therapy in 98.2%, 66.3%, 41.5%, and 60.9%, respectively. Anthracyclines were administered to 30.1% and 5.8% received trastuzumab. The median interval from BC diagnosis to ETT was 8.7 [4.5, 14.3] years.

### 3.3 Exercise testing indications and results

All patients were referred for clinical indications, with chest pain being more frequent among controls (32.1% vs 43.8%, *P* = 0.0004) (Table S2). Exercise‐induced symptoms and ECG changes were similar across groups. Overall, self‐reported fatigue was the most common reason for terminating exercise and was more frequent in BC cohort compared to controls (67.2% vs 58%, *P* = 0.006) (Table S2). Frequency of positive ETTs was similar between groups (Table S2).

### 3.4 Primary endpoints

BC cohort had a significantly higher resting HR than controls (71 ± 12 vs 69 ± 12 bpm, *P* = 0.025; Table 2). HRR at 1 minute after termination of exercise was significantly reduced in BC cohort relative to controls (24 ± 13 vs 27 ± 15 bpm, *P* = 0.0016; Table 2).

**Table 2:** Study end‐points

End‐points	Breast cancer (n = 448)	Controls (n = 448)	*P* value
Elevated resting heart rate			
HR at rest, mean (SD), bpm	71 ± 12	69 ± 12	0.025
Elevated resting HR ≥80 bpm, n (%)	106 (23.7)	76 (17.0)	0.013
Elevated heart rate recovery			
HR at peak exercise, mean (SD), bpm	146 ± 22	142 ± 23	0.018
HR at 1 min recovery, mean (SD), bpm	122 ± 20	116 ± 20	<0.0001
HRR at 1 min, mean (SD), bpm	24 ± 13	27 ± 15	0.0016
Abnormal HRR, n (%)	115 (25.9)	91 (20.3)	0.048
Secondary end‐point			
METs achieved, mean (SD)	9.0 ± 2.9	8.7 ± 2.7	0.22

HR, heart rate; HRR, heart rate recovery; METs, metabolic equivalents.

**Figure 1:**
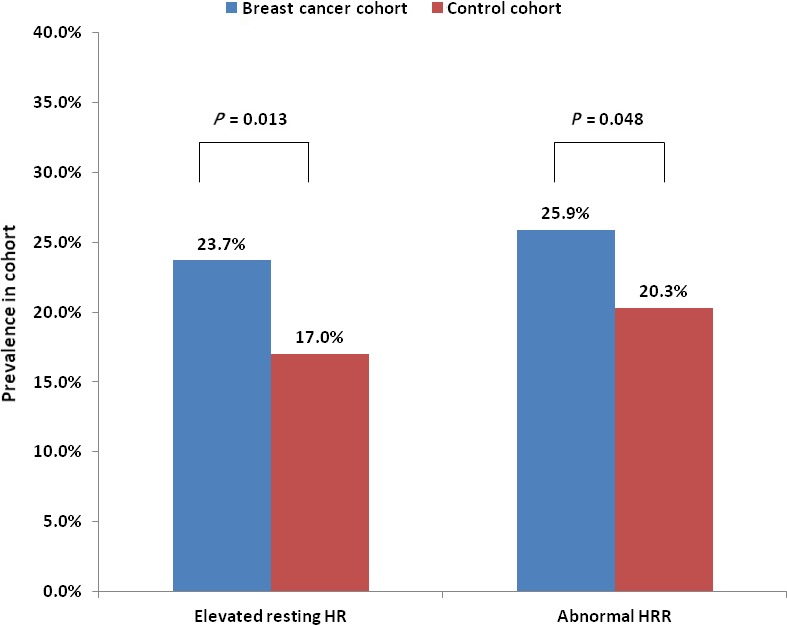
Prevalence of elevated resting heart rate (HR) and abnormal heart rate recovery (HRR) in breast cancer (BC) and control cohorts

Elevated resting HR (23.7% vs 17.0%, *P* = 0.013) and abnormal HRR (25.9% vs 20.3%, *P* = 0.048) were more prevalent in BC cohort compared to controls (Figure 1). BC conferred an increased risk of both elevated resting HR (OR 1.51, 95% CI 1.09‐2.11, *P* = 0.01) and abnormal HRR (OR 1.37, 95% CI 1.0‐1.87, *P* = 0.048). This association remained significant after adjustment for Morise score, AV nodal blocking agents, and result of ETT (elevated resting HR: adjusted OR 1.53, 95% CI 1.08‐2.16, *P* = 0.02; abnormal HRR: adjusted OR 1.75, 95% CI 1.23‐2.48, *P* = 0.002). Examination of these parameters as a function of time from cancer treatment showed no significant decay in their frequency, suggesting that deconditioning shortly after cancer treatment was not the primary cause of these abnormalities (Table S3).

### 3.5 Secondary endpoints

There were no significant differences in mean exercise duration or METs achieved between the two groups (Table 2).

### 3.6 Functional significance of elevated resting HR and abnormal HRR

**Figure 2:**
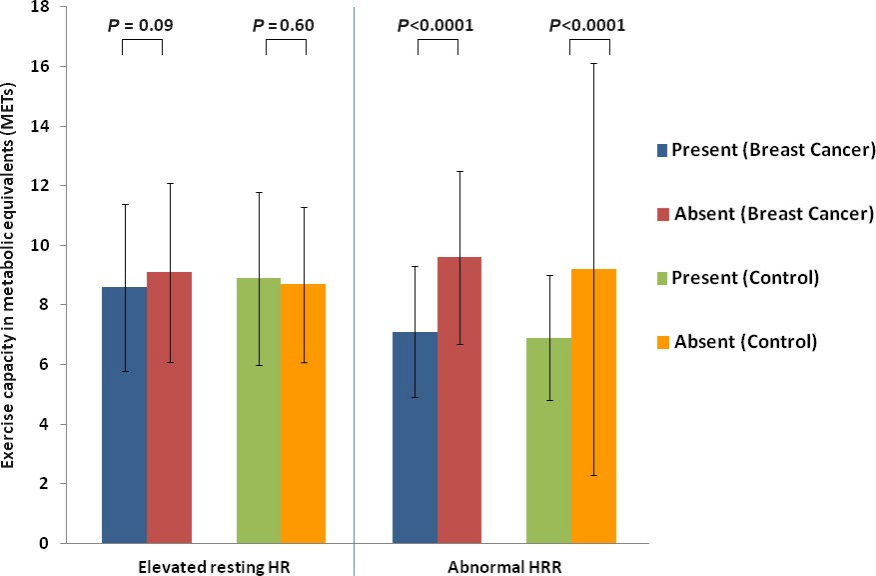
Unadjusted comparison of exercise capacity among breast cancer patients and age‐ and sex‐matched control patients based on the presence or absence of an elevated resting heart rate (HR), and abnormal heart rate recovery (HRR)

In unadjusted analyses, abnormal HRR, but not elevated resting HR, was associated with significantly decreased exercise capacity in BC cohort and controls (Figure 2). After adjustment for age, BMI, cardiovascular risk factors, statins, AV nodal blocking agents, and result of ETT, both elevated resting HR (−0.9 METs (SE 0.3); *P* = 0.0003) and abnormal HRR (−1.3 METs (SE 0.3); *P* < 0.0001) were associated with significantly decreased exercised capacity in BC cohort. Similarly, in adjusted analyses, both elevated resting HR (−0.6 METs (SE 0.3); *P* = 0.03) and abnormal HRR (−0.6 METs (SE 0.3); *P* = 0.02) were also associated with decreased exercise capacity in controls. Alternative categorization strategies for resting HR (as described in Methods above) identified similar reductions in exercise capacity for higher categories of resting HR (data not shown).

### 3.7 Prognostic significance of elevated resting HR and abnormal HRR

A total of 30 (6.7%) BC and 9 (2.0%) control patients died over a median follow‐up of 3.5 [1.9, 5.4] years. Specific causes of death are shown in Table S4. There was only one cardiovascular death in each group. There was no association between elevated resting HR and all‐cause mortality in either cohort (Table 3). Similarly, no associations with all‐cause mortality were identified when alternative categorization strategies for resting HR were employed in sensitivity analyses (data not shown). While abnormal HRR was associated with increased all‐cause mortality among controls, this only trended toward significance in BC cohort (adjusted hazard ratio 2.06 (95% CI 0.95‐4.44, *P* = 0.07)) (Table 3).

**Table 3:** Association of elevated resting heart rate and abnormal heart rate recovery with all‐cause mortality in breast cancer cohort and age‐ and sex‐matched controls

	Breast cancer		Controls		*P* value for interaction
	Unadjusted HR (95% CI) *P* value	Adjusted HR^a^(95% CI) *P* value	Unadjusted HR (95% CI) *P* value	Adjusted HR^a^ (95% CI) *P* value	
Elevated Resting HR	1.39 (0.65, 2.97) *P* = 0.40	1.32 (0.61, 2.89) *P* = 0.48	0.73 (0.09, 5.91) *P* = 0.77	0.97 (0.12, 7.95) *P* = 0.98	0.57
Abnormal HRR	1.87 (0.90, 3.89) *P* = 0.10	2.06 (0.95, 4.44) *P* = 0.07	6.63 (1.59, 27.77) *P* = 0.01	5.11 (1.16, 22.48) *P* = 0.03	0.12

HR, heart rate; HRR, heart rate recovery.

a Adjusted for age, AV nodal blocking agents, Morise risk score, and result of ETT.

### 3.8 Association between specific cancer therapies and elevated resting HR and abnormal HRR

In exploratory analyses, only prior radiation (adjusted OR 1.54, 95% CI 1.09‐2.17, *P* = 0.02) and hormonal therapy (adjusted OR 1.52, 95% CI 1.08‐2.16, *P* = 0.02) were associated with an increased likelihood of elevated resting HR No associations were seen between different treatment modalities and abnormal HRR (Table 4).

**Table 4:** Unadjusted and adjusted measures of association for various breast cancer treatments and elevated resting heart rate and abnormal heart rate recovery

Cancer therapy	Elevated resting HR				Abnormal HRR			
	Unadjusted OR (95% CI)	*P* value	Adjusted OR^a^(95% CI)	*P* value	Unadjusted OR (95% CI)	*P* value	Adjusted OR^a^(95% CI)	*P* value
Adjuvant Chemotherapy	1.40 (0.96, 2.05)	0.08	1.29 (0.87, 1.91)	0.20	1.13 (0.78, 1.65)	0.52	1.34 (0.90, 2.01)	0.15
Anthracyclines	1.53 (1.01, 2.34)	0.05	1.35 (0.87, 2.08)	0.18	1.04 (0.68, 1.60)	0.86	1.40 (0.88, 2.21)	0.15
Radiation Therapy	1.47 (1.05, 2.05)	0.03	1.54 (1.09, 2.17)	0.02	1.16 (0.84, 1.61)	0.37	1.12 (0.79, 1.58)	0.53
Hormonal Therapy	1.57 (1.12, 2.21)	0.01	1.52 (1.08, 2.16)	0.02	1.26 (0.90, 1.75)	0.17	1.33 (0.94, 1.89)	0.11

AV, atrio‐ventricular, ETT, exercise treadmill test.

a Adjusted for Morise score, AV node blocking drugs, and result of ETT.

## 4 DISCUSSION

Our results demonstrate that among women clinically referred for ETT, elevated resting HR and abnormal HRR are more prevalent in women with a history of BC compared to cancer‐free, age‐matched controls. Importantly, the presence of an elevated resting HR or abnormal HRR was associated with decreased exercise capacity. As observed in the general population, abnormal HRR predicted increased all‐cause mortality in control patients. While not statistically significant, there was a trend toward increased mortality in BC cohort with abnormal HRR.

Prior studies have shown decreased exercise capacity across the BC survivorship continuum. Peak oxygen consumption, a measure of aerobic power, is substantially lower in BC survivors compared to healthy women.^7^, ^25^, ^26^ In this study, we explored mechanisms underlying this decreased functional capacity in patients with a history of BC who were clinically referred for ETT. In our study, there was no difference in mean exercise capacity between the BC cohort and controls, even though the BC cohort had fewer cardiovascular risk factors than controls.

We have previously shown that survivors of Hodgkin lymphoma, treated with mediastinal radiation, have increased prevalence of an elevated resting HR and abnormal HRR compared to age‐ and gender‐matched controls.^18^ Furthermore, in these Hodgkin survivors, an elevated resting HR or abnormal HRR were associated with decreased exercise capacity.^18^ In the current study, we examined whether these parameters were more prevalent in a BC cohort and whether they contribute to the decreased exercise tolerance observed in these patients. As with Hodgkin survivors, we observed an increased prevalence of these abnormalities in our BC cohort compared to cancer‐free controls. Furthermore, we found that that the presence of either an elevated resting HR or abnormal HRR was associated with significantly decreased exercise capacity.

Multiple mechanisms can be postulated to explain the higher prevalence of resting HR and abnormal HRR across the BC survivorship continuum. Direct sympatho‐vagal injury due to cancer or its treatments, subclinical left ventricular dysfunction or diastolic dysfunction due to cancer therapy, as well as indirect effects of cancer‐associated lifestyle perturbations, should be considered. While we were unable to demonstrate a consistent association between different cancer treatments and the presence of an elevated resting HR or abnormal HRR, both markers of cardiac dysautonomia, previous studies have shown that BC treatment with anthracyclines and paclitaxel were each associated with impaired autonomic function, especially during therapy.^27^, ^28^ The lack of an association between anthracycline exposure and elevated HR and HRR in our study, which consisted primarily of survivors, may reflect diminished alterations in autonomic markers with time from chemotherapy. In addition, psychosocial stress, anxiety, depression, sleep disturbances, sarcopenic weight gain, and low cardiorespiratory fitness are prevalent in BC patients and each of these has been implicated in the pathogenesis of autonomic dysfunction.^28^, ^29^, ^30^ Hormonal therapy with tamoxifen has been associated with weight gain and aromatase inhibitors with musculoskeletal discomfort. These side effects may alter exercise tolerance and thus may affect cardiorespiratory fitness in BC patients.

It is well established that elevated resting HR and abnormal HRR predict increased cardiovascular and all‐cause mortality in healthy individuals and patients with cardiovascular diseases.^10^, ^31^, ^32^ We chose resting HR ≥80 bpm based on data from the National FINRISK study involving 11 334 healthy women, where resting HR ≥82 bpm was associated with a hazard ratio of 1.6 (95% CI 1.2‐2.1) for cardiovascular mortality and 5.6 (95% CI 4.8‐6.5) for all‐cause mortality.^14^ This risk remained significant even after adjustment for traditional cardiovascular risk factors. A recent meta‐analysis of 46 studies involving 1 246 203 patients similarly showed that resting HR ≥80 bpm conferred a relative risk of 1.33 (95% CI 1.19‐1.47) for cardiovascular mortality and 1.45 (95% CI 1.34‐1.57) for all‐cause mortality, independent of cardiovascular risk factors.^13^ Elevated resting HR and decreased HR variability predicted survival in a small study of 57 patients with advanced BC.^33^ Similarly, a retrospective study of 4786 patients with stage I‐III BC found that patients in the highest quintile of resting HR (≥85 bpm) had significantly higher all‐cause mortality than those in the lowest quintile (≤67 bpm), after adjusting for other prognostic factors.^15^ Unlike these prior studies, we evaluated predominantly survivors rather than women with active BC. We were unable to demonstrate a significant association between resting HR or abnormal HRR and all‐cause mortality across the BC survivorship continuum. This is likely due to the relatively short follow‐up (3.5 [1.9, 5.4] years) and small number of deaths (n = 30) in our study.

### 4.1 Limitations

This study has several potential limitations. Patients were clinically referred for ETT and had a high prevalence of cardiovascular risk factors and ischemic heart disease. Thus, it is possible that elevated resting HR and abnormal HRR were more prevalent in this study compared to general BC patients. However, given our retrospective study design, systematic exercise data were unavailable in patients with no clinical indication for ETT. Furthermore, despite a higher burden of cardiovascular risk factors and ischemic heart disease in control patients, elevated resting HR and abnormal HRR were more prevalent in the BC cohort, suggesting that BC predisposes patients to these abnormalities, independent of cardiovascular risk factors. Echocardiographic data were available in a subset of patients. Mean LVEF was normal in both cohorts, but we cannot exclude the possibility of subclinical LV systolic or diastolic dysfunction as a contributor to these abnormalities. Additionally, psychosocial factors may also play a role and were not captured. Lastly, given the small number of deaths in this study cohort, we were inadequately powered to detect an association between elevated resting HR or abnormal HRR and all‐cause mortality in the BC cohort. However, we did demonstrate an association between abnormal HRR and all‐cause mortality in control patients, with a trend in the BC cohort. Similarly, the two cardiovascular deaths observed in our cohort did not allow us to examine the association between the primary outcome measures and cardiovascular mortality.

## 5 CONCLUSIONS

In conclusion, women across the BC survivorship continuum referred for ETT exhibit an increased prevalence of elevated resting HR and abnormal HRR after exercise compared to cancer‐free, age‐ and sex‐matched controls. Importantly, the presence of an elevated resting HR or abnormal HRR is associated with reduced exercise capacity. Studies involving strategies that modulate resting heart rate and abnormal heart rate recovery may lead to improved functional capacity and quality of life for women following a diagnosis of BC.

## CONFLICT OF INTEREST

AN reports a research grant from Amgen, Inc and is a consultant for Takeda Oncology outside of submitted work. JDG reports a research grant from Amgen, Inc outside of submitted work. TGN reports personal fees from Takeda Oncology and Parexel outside of submitted work. MDC reports research grants from Spectrum Dynamics, and personal fees from Sanofi, and General Electric outside of submitted work. MRM reports personal fees from Abbott, Medtronic, NupulseCV, Portolam Mesoblast, and Bayer outside of submitted work. SSM, DP, SG, JH, AHP, and LWJ have no relevant disclosures.
