# Supplementary material for: Case‐control study of heart rate abnormalities across the breast cancer survivorship continuum
Source: Cancer Med. 2018 Dec 21;8(1):447–54. doi: 10.1002/cam4.1916 (PMC6346251; doi:10.1002/cam4.1916)
Supplement: Supplementary file 1 [file CAM4-8-447-s001.docx]

**SUPPLEMENTAL MATERIAL:**

**Supplemental Table 1: Oncologic characteristics of the breast cancer cohort.**

| Interval from diagnosis to ETT, median (IQR), years | 8.7 (4.5, 14.3) |
| --- | --- |
| Laterality of breast cancer:  Right breast, n (%)  Left breast, n (%)  Bilateral disease, n (%)  Unknown, n (%) | 204 (45.5)  227 (50.7)  15 (3.4)  2 (0.4) |
| Estrogen receptor status:  Negative, n (%)  Positive, n (%)  Unknown, n (%) | 63 (14.1)  283 (63.2)  102 (22.8) |
| Progesterone receptor status:  Negative, n (%)  Positive, n (%)  Unknown, n (%) | 72 (16.1)  224 (50.0)  152 (33.9) |
| Her2 receptor status:  Negative, n (%)  Positive, n (%)  Unknown, n (%) | 197 (44.0)  58 (13.0)  193 (43.1) |
| Adjuvant Chemotherapy, n (%) | 186 (41.5) |
| Anthracycline, n (%) | 135 (30.1) |
| ^a^Anthracycline dose, median (IQR), mg/m^2^ | 240 (240, 240) |
| Trastuzumab, n (%) | 26 (5.8) |
| Surgery, n (%) | 440 (98.2) |
| Radiation therapy, n (%)  Right breast radiation, n (%)  Left breast radiation, n (%) | 297 (66.3)  156 (34.8)  169 (37.7) |
| Hormone therapy, n (%) | 273 (60.9) |
| Stage at diagnosis, n (%) | 0=30 (6.7)  I=176 (39.3)  II=118 (26.3)  III=21 (4.7)  IV=3 (0.7)  Unknown=100 (22.3) |
| ^a^Dose of anthracycline could not be determined for 17 of 135 (12.6%) women who received anthracyclines.  ETT = exercise treadmill test | |

**Supplemental Table 2: Indications for exercise treadmill testing and results.**

|  | **Breast Cancer**  **(n=448)** | **Controls**  **(n=448)** | **p value** |
| --- | --- | --- | --- |
| **Indication for ETT**  Chest pain, mean, n (%)  Dyspnea, n (%)  Arrhythmia, n (%)  Other, n (%) | 144 (32.1)  90 (20.1)  49 (10.9)  165 (36.8) | 196 (43.8)  92 (20.5)  42 (9.4)  118 (26.3) | 0.0004  0.93  0.51  0.0009 |
| Exercise duration, minutes | 7.4±2.9 | 7.3±2.9 | 0.39 |
| **ECG changes during ETT**  None, n (%)  ST depression, n (%)  ST elevation, n (%)  Other changes, n (%) | 263 (58.7)  44 (9.8)  1 (0.2)  142 (31.7) | 233 (52.0)  57 (12.7)  1 (0.2)  156 (34.8) | 0.05  0.21  1.00  0.36 |
| **Reason for terminating test**^*^  Chest pain, n (%)  Dyspnea, n (%)  Fatigue, n (%)  Hypertensive response, n (%)  Other reason, n (%) | 8 (1.8)  168 (37.5)  301 (67.2)  3 (0.7)  58 (13.0) | 14 (3.1)  176 (39.3)  260 (58.0)  5 (1.1)  76 (17.0) | 0.28  0.63  0.006  0.73  0.11 |
| **Result of ETT**  Negative, n (%)  Positive, n (%)  Inconclusive, n (%) | 371 (82.8)  42 (9.4)  35 (7.8) | 363 (81.0)  57 (12.7)  28 (6.3) | 0.21 |
| Cumulative percentages may exceed 100% as categories are not mutually exclusive.  ETT = exercise tolerance test. | | | |

**Supplemental Table 3: Frequency of elevated resting heart rate and abnormal heart rate recovery across the breast cancer survivorship continuum according to duration from cancer diagnosis/treatment.**

|  | **<5 years** | **5-10 years** | **>10 years** | **p value** |
| --- | --- | --- | --- | --- |
| **Elevated resting HR, n (%)** | 39/131 (29.8) | 28/125 (22.4) | 39/192 (20.3) | 0.06 |
| **Abnormal HRR, n (%)** | 33/131 (25.2) | 42/125 (33.6) | 41/192 (21.4) | 0.32 |
| HR = heart rate, HRR = heart rate recovery | | | | |

**Supplemental Table 4: Frequency and causes of death among breast cancer cohort and age- and sex-matched control patients.**

|  | **Breast cancer (n=448)** | **Controls (n=448)** |
| --- | --- | --- |
| **All cause death, n (%)** | 30 (6.7) | 9 (2.0) |
| **Cancer death, n (%)** | 24 (5.4) | 7 (1.6) |
| **Cardiovascular death, n (%)** | 1 (0.2) | 1 (0.2) |
| **Hemorrhagic stroke, n (%)** | 1 (0.2) | 0 (0) |
| **Other causes, n (%)** | 4 (0.9) | 1 (0.2) |
